# Assessing the Contribution of the Neutral Blocks in DNA/Block-Copolymer Polyplexes: Poly(acrylamide) vs. Poly(ethylene Oxide)

**DOI:** 10.3390/molecules28010398

**Published:** 2023-01-03

**Authors:** Renata Mello Giona, Letícia Vitorazi, Watson Loh

**Affiliations:** 1 LaMaFI—Laboratório de Materiais e Fenômenos de Interface, Chemistry Department, Universidade Tecnológica Federal do Paraná (UTFPR), Medianeira, Curitiba 85884-000, Paraná (PR), Brazil; 2 Institute of Chemistry, Universidade Estadual de Campinas (UNICAMP), Caixa Postal 6154, Campinas 13083-970, São Paulo State (SP), Brazil; 3 Laboratório de Materiais Poliméricos, EEIMVR, Universidade Federal Fluminense, Volta Redonda 27255-125, Rio de Janeiro (RJ), Brazil

**Keywords:** polyplex, coacervation, DNA, block copolymers, ITC, polyelectrolytes

## Abstract

The interaction of DNA with different block copolymers, namely poly (trimethylammonium chloride methacryloyoxy)ethyl)-block-poly(acrylamide), i.e., (PTEA)-*b*-(PAm), and poly (trimethylammonium chloride methacryloyoxy)ethyl)-block-poly(ethylene oxide), i.e., (PTEA)-*b*-(PEO), was studied. The nature of the cationic block was maintained fixed (PTEA), whereas the neutral blocks contained varying amounts of acrylamide or (ethylene oxide) units. According to results from isothermal titration microcalorimetry measurements, the copolymers interaction with DNA is endothermic with an enthalpy around 4.0 kJ mol^−1^ of charges for (PTEA)-*b*-(PAm) and 5.5 kJ mol^−1^ of charges for (PTEA)-*b*-(PEO). The hydrodynamic diameters of (PTEA)-*b*-(PEO)/DNA and (PTEA)-*b*-(PAm)/DNA polyplexes prepared by titration were around 200 nm at charge ratio (Z_+/−_) < 1. At Z_+/−_ close and above 1, the (PTEA)_50_-*b*-(PAm)_50_/DNA and (PTEA)_50_-*b*-(PAm)_200_/DNA polyplexes precipitated. Interestingly, (PTEA)_50_-*b*-(PAm)_1000_/DNA polyplexes remained with a size of around 300 nm even after charge neutralization, probably due to the size of the neutral block. Conversely, for (PTEA)_96_-*b*-(PEO)_100_/DNA polyplexes, the size distribution was broad, indicating a more heterogeneous system. Polyplexes were also prepared by direct mixture at Z_+/−_ of 2.0, and they displayed diameters around 120–150 nm, remaining stable for more than 10 days. Direct and reverse titration experiments showed that the order of addition affects both the size and charge of the resulting polyplexes.

## 1. Introduction

Polyelectrolyte self-assemblies are present in many biological systems, and it is interesting to develop artificial self-assemblies that mimic naturally occurring assembly structures [1,2]. For instance, DNA is a highly negatively charged polyelectrolyte and therefore adopts an extended coil conformation in dilute solutions [3,4]. Considering that several diseases can be treated using gene therapy, numerous efforts have been applied to develop efficient DNA delivery vectors. Viruses can transport genetic material into the nucleus once they go through endocytosis to the cell. However, although cationic non-viral vectors are in general more cytotoxic and less efficient, due to safety reasons, there is an increased interest in the development of nonviral vectors using cationic agents [5], for instance, the surfactants CTAB [6], DODAB and DODAC [7], ester-based cationic gemini surfactants [8], and synthetic polymers such as poly(ethyleneimine) [9,10,11], polylysine [12,13], poly(glycoamidoamine) [14], poly(amide triazole) [15] and poly(amido amine) dendrimer, PAMAM [16,17,18]. Furthermore, there are current human clinical trials using DNA-based vaccines that target various types of diseases such as autoimmune infectious and against coronavirus, for example [19]. Therefore, it is of great interest to develop carrier materials to improve the systemic release of DNA in the body [19].

Cationic molecules bind to the negatively charged DNA backbone, forming a complex in which the DNA backbone is compacted [20,21,22]. Several functional groups are interesting to investigate regarding their attachment to macromolecules, such as trimethylammonium [4] and guanidinium [23], among others. In fact, ammonium groups play an important role in the efficiency of genetic material transport with cationic polymers because they form condensed particles with DNA, sometimes referred to as polyplexes [24]. 

Many studies seek to design and select appropriate DNA carriers and aim at elucidating the features that govern the interaction between the DNA and the cationic agent and how the biological activity of these polyplexes is preserved. Chen and co-workers synthesized a triblock copolymer of polylysine-*b*-polyleucine-*b*-polylysine with different block lengths as free cationic chains and used natural protamine to condense DNA, with results showing that these triblock copolypeptides are able to increase the gene transfection efficiency by a factor of approximately 10^4^ times compared with the use of the polyplexes without the free cationic chains [22]. Jung and co-workers prepared several block copolymers in which the cationic block was fixed as poly(N-(2-aminoethyl) methacrylamide) (PAEMA) with varying hydrophilic blocks including poly(ethylene glycol), poly(oligo(ethylene glycol) methyl ether methacrylate), and poly(2-deoxy-2-methacrylamido glucopyranose). According to their results, the nature of the hydrophilic block plays an important role in determining the structures of pDNA−diblock complexes [25]. However, despite the large number of studies involving the interaction of DNA with cationic polyelectrolytes [3,12,21,22,23,24,25,26,27,28,29,30,31,32,33,34], the thermodynamics of the polyplexes formation is not completely understood yet, especially concerning contributions from interaction forces other than electrostatic ones. 

Interactions between macromolecules have been extensively characterized by isothermal titration calorimetry (ITC), a technique that enables the direct measurement of the heat exchange involved when one species is titrated into another, providing information on the extent of interaction as well as on enthalpy changes associated with the binding process. Therefore, ITC is an outstanding tool not only to monitor binding processes but also to produce a complete thermodynamic profile of the system [35,36,37].

**Scheme 1:**
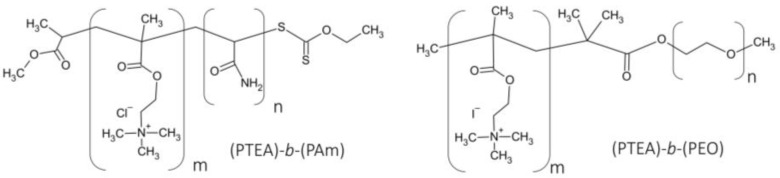
Chemical structures of block copolymers used in this study: (PTEA)_50_-*b*-(PAm)_50_, (PTEA)_50_-*b*-(PAm)_200_, (PTEA)_50_-*b*-(PAm)_1000_, and (PTEA)_96_-*b*-(PEO)_100_.

In this paper, we report the preparation of three different block copolymers containing the same cationic and neutral portions but with different neutral block lengths, namely (trimethylammonium chloride methacryloy-oxyethyl)-block-poly(acrylamide), (PTEA)_50_-*b*-(PAm)_50_, and (PTEA)_50_-*b*-(PAm)_200_ and (PTEA)_50_-*b*-(PAm)_1000._ Another copolymer containing poly(ethylene oxide) as the neutral block, (PTEA)_96_-*b*-(PEO)_100_, was also used, as shown in Scheme 1. In this approach, the block copolymers used contained the same cationic block, allowing the assessment of the influence of the type and size of their neutral blocks on the formation of DNA polyplexes. These polyplexes were characterized by isothermal titration microcalorimetry (ITC), zeta potential and dynamic light-scattering measurements, and via circular dichroism spectroscopy.

## 2. Results and Discussion

Three block copolymers were synthesized with varying sizes of their polyacrylamide blocks and with the same cationic block. The intent was to verify the influence of their neutral acrylamide blocks on the stabilization of the complex formed through the interaction between the cationic portion of the copolymer and the negatively charged DNA. In addition, another block copolymer was modified to contain the same cationic block and with a PEO neutral block instead of PAm, namely (PTEA)_96_-*b*-(PEO)_100_. Table 1 summarizes the copolymers prepared as well as the expected size of each block considering the quantity of reactants used in the synthesis of (PTEA)-*b*-(PAm). Their dispersity values (M_w_/M_n_) were calculated using GPC experimental data. The copolymer (PDMAEMA)_96_-*b*-(PEO)_100_ was modified in order to obtain the block copolymer (PTEA)_96_-*b*-(PEO)_100_, and its composition was provided by the supplier.

The interaction of copolymers with DNA was investigated using ITC, DLS, zeta potential, and circular dichroism measurements, varying the charge ratio between polymer and DNA (Z_+/−_ ratio), that is, the ratio between number of positive charge equivalents of the polymer and the negative charge equivalents of the nucleic acid. We decided to work in low-ionic-strength media because the presence of electrolytes might increase the size of polyplexes due to charge screening effects [38].

**Figure 1:**
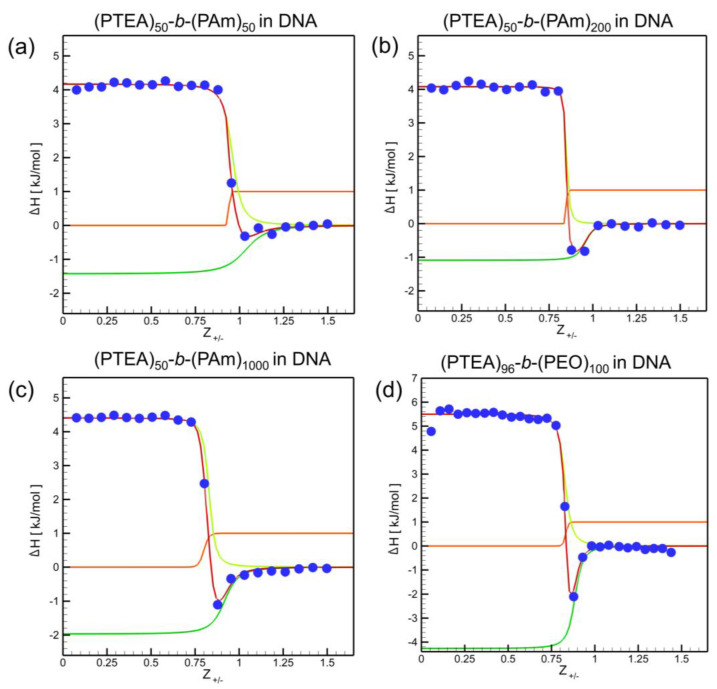
Binding enthalpy for titration (**a**) PTEA_50_-*b*-PAm_50_, (**b**) PTEA_50_-*b*-PAm_200_, (**c**) PTEA_50_-*b*-PAm_1000_, and (**d**) PTEA_96_-*b*-PEO_100_ with DNA, as a function of their charge ratios (Z_+/−_).

Calorimetric titrations of DNA solutions with (PTEA)-*b*-(PAm) were performed to investigate the energetics of binding. Figure 1a–c show the integrated heats of reaction plotted against *Z*_+/-_. For the addition of (PTEA)-*b*-(PAm) to DNA, in the presence of NaCl 5.0 mM, the enthalpy of interaction (ΔH) is around 4.0 kJ mol^−1^ expressed per mole of added copolymer in each injection and is constant and endothermic up to Z_+/−_ value around 0.85. This means that this complex formation is entropically driven, probably due to the release of water molecules and counterions that occurs when the opposite charged chains bind. The order of magnitude of these enthalpy changes agrees with previous determination for the interaction of other oppositely charged polymers, suggesting a general behavior [7,38]. Similarly, for (PTEA)_96_-*b*-(PEO)_100_, ITC experiments showed that the interaction is also endothermic with larger values of enthalpy of binding (ΔH) around 5.5 kJ mol^−1^ expressed per mole of added copolymer in each injection when compared to the (PTEA)-*b*-(PAm) copolymers and that it remains constant up to Z_+/−_ of 0.83 (Figure 1d).

In mixtures with Z_+/−_ below 1.0, DNA in excess interacts with the complexes to produce negatively charged and kinetically stable aggregates. Subsequently to the abrupt decrease in ΔH, after charge neutralization, as Z_+/−_ increases, the observation of ΔH values close to zero indicates that the added polycation does not interact with the structures formed [38]. As soon as a charge ratio of around 1.0 is reached, and before ΔH is close to zero and constant, an endothermic peak occurs. Previous results reported that as the charge ratio approaches stoichiometry, the repulsion decreases, and the complexes gradually transform into a larger complex coacervate by stepwise aggregation until nanophase separation [39]. These findings corroborate with the previously proposed two-step process, in which the first process is related to the formation of complexes of highly charged polyelectrolyte of size around 100 nm, and the second process is the transition of a coacervate phase [38,40]. On the other hand, the complexation between quaternized poly(2-(dimethylamino)ethyl methacrylate-*b*-laurylmethacrylate-*b*-(oligo ethylene glycol) methacrylate) amphiphilic triblock terpolymer micelles follows a two-step interaction process only for the terpolymer/short DNA micellar polyplexes (113 bp), and a only a single process is observed when complexation occurs with the 2000 bp DNA [41]. 

These curves (Figure 1) display features already reported for other electrostatic binding processes involving surfactants and polymers, such as the positive values for enthalpy of binding and the appearance of a bump in the ITC curves close to charge neutrality [38,40]. The latter feature has been ascribed to a two-stage association process in which ion pairs are formed initially, and closer to charge neutrality, the whole system rearranges to form a coacervate that eventually phase separates. In order to better understand the association thermodynamics in the two processes, A and C, observed (with A standing for aggregation and C coacervation), fitting of the calorimetry data was performed using the modified version of the Multiple Non-Interacting Sites (MNIS) model as previously proposed [38]. The interaction between both macromolecules is associated with a binding enthalpy (Δ*H_b_*), a binding constant *K*_b_, and a reaction stoichiometry *n*, which is the number of binding sites assuming that they do not interact one with another [38]. As one macromolecule is titrated into the other, the heat exchange is quantified by the derivative of the heat related to the incremental addition of a small amount of macromolecule (ligand). Thus, using the charge ratio Z_+/−_ to associate the concentration of both macromolecules, the enthalpy can be defined by Equation (1):

(1) $$\Delta H\left(Z,n,r\right)=\frac{1}{2}\Delta H_{b}\left(1+\frac{n-Z-r}{\sqrt{{\left(n+Z+r\right)}^{2}}-4Zn}\right)$$

where $r=1/K_{b}\left[M\right]$, and [M] is the molar concentration of the macromolecule in the cell. To account for the two-step titration, one assumes that the heat exchange is the sum of the two contributions, $\Delta H_{A}\left(Z,n_{A},r_{A}\right)$ for the polyplexes binding and $\Delta H_{C}\left(Z,n_{C},r_{C}\right)$ for the coacervation, where A stands for aggregates and C for coacervates formation. Thus, the total enthalpy change for the titration is the sum of the binding enthalpies for each process, $\Delta H_{b}^{A}$ and $\Delta H_{b}^{C}$, namely aggregation and coacervation, respectively, expressed as:

(2) $$\Delta H\left(Z\right)=\Delta H_{A}\left(Z,n_{A},r_{A}\right)+\alpha \left(Z\right)\Delta H_{C}\left(Z,n_{C},r_{C}\right),$$

where the function α(Z) is the fraction of the coacervate phase formed at a specific charge ratio Z_+/−_ and defined as follows:

(3) $$\alpha \left(Z\right)={\left(1+\exp\left(-\frac{\left(Z-Z_{0}\right)}{\sigma }\right)\right)}^{-1},$$

From the binging enthalpy ($\Delta H_{b}$), the stoichiometry (n), and the binding constant ($K_{b}$), the changes in free energy (ΔG) and in entropy (ΔS) of the interaction can be calculated as follows:

(4) $$\Delta G=-RTlnK_{b},$$

(5) $$\Delta S=\frac{\left(\Delta H_{b}-\Delta G\right)}{T},$$

These parameters were obtained considering the molar composition in terms of charge equivalents (for this system, one charge equals one mer). Table 2 presents the thermodynamic parameters obtained by the fitting of the ITC curves for processes A and C, as described above.

Further analysis of the thermodynamics of process C shows that the coacervation/precipitation is exothermic for the addition of all the block copolymers in DNA. The enthalpy values were similar for all the polyplexes, with exception of PTEA_96_-*b*-PEO_100_ with DNA, whose binding is found to be more energetic. 

Moreover, the entropy changes are positive and in the range of 103–155 J mol^−1^ K^−1^, confirming an entropically driven process because enthalpy values are positive, which is in accordance with previous studies [38,42]. The entropy values of process A are greater than those of process C, as is for Gibbs free energy. In addition, the enthalpy values that were positive in process A became negative in process C although both processes are controlled by entropy. Process A occurs in a stoichiometry below that of process B, with the first continuing up to Z = 0.83–0.96, while the second occurs at Z = 0.89–1.04, suggesting that coacervation only takes place when most of the charges are neutralized. With respect to K, the values obtained in the present study are larger than values reported in previous studies [43].

**Figure 2:**
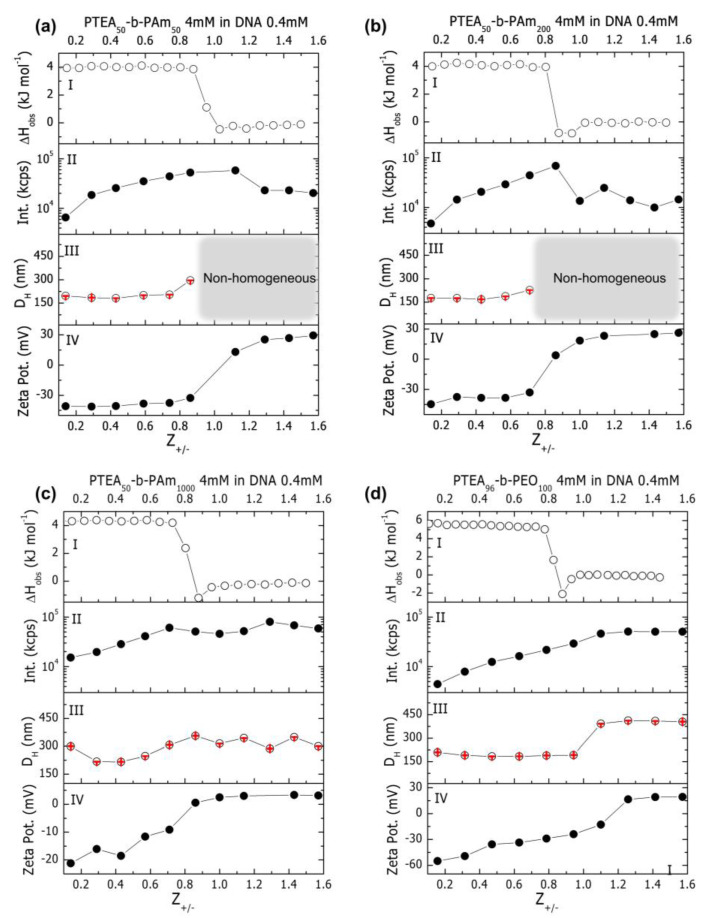
Comparison of ITC data with DLS results: intensity of scattering; hydrodynamic diameter (Z_average_) with PDI values indicated as red bars; and zeta potential values for titration of DNA with (PTEA)_50_-*b*-(PAm)_50_ (**a**), (PTEA)_50_-*b*-(PAm)_200_ (**b**), (PTEA)_50_-*b*-(PAm)_1000_ (**c**), and (PTEA)_96_-*b*-(PEO)_100_ (**d**).

In order to shed light on the structural changes upon the different complexation processes, a DLS investigation was performed and compared with the calorimetry data as in Figure 2a–d, where I displays the calorimetric data presented above, II is the scattering intensity data, III the hydrodynamic diameters with polydispersity index values, and IV shows the zeta potential data.

The scattering intensity data show that there is an increase in scattering intensity with an increase in the charge ratio up to the value of Z_+/−_ around 0.8. Then, there is a slight decrease in scattering intensity for polymers with PAm_50_ and PAm_200_ blocks for higher charge ratios. This behavior is different for the system composed by PAm_1000_, where these values remain somewhat constant for intermediate Z_+/−_ values with a slight increase trend. Studies show that this variation in intensity is a typical feature associated with the formation of coacervates or precipitates during these titrations; when the intensity decreases after coacervation or precipitation, this indicates that larger aggregates are formed that phase separate from the solution and stop scattering light [38].

**Figure 3:**
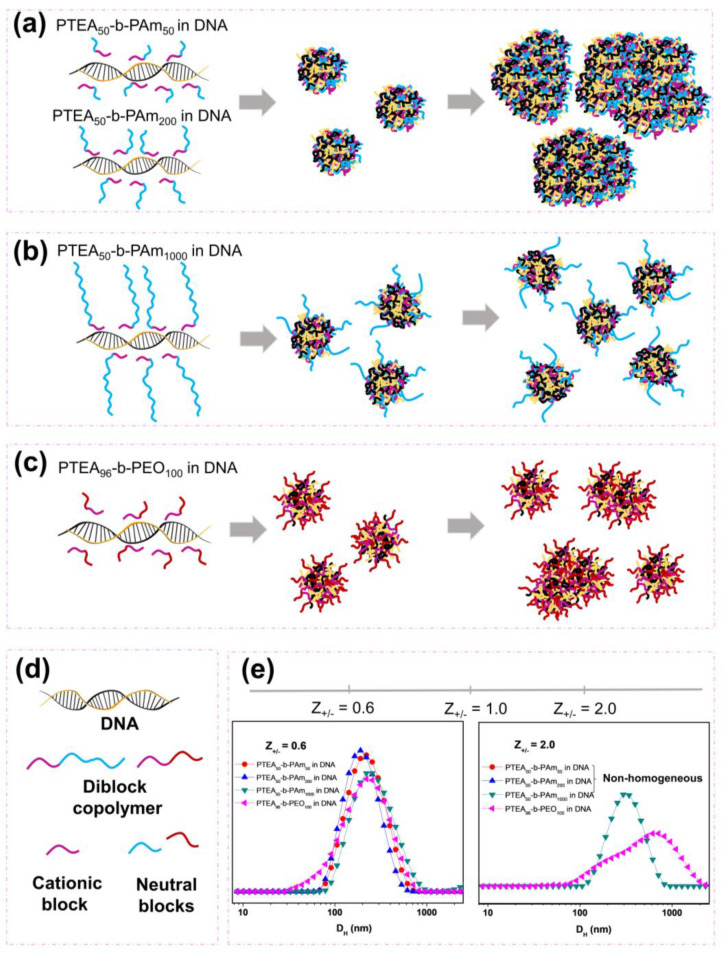
Illustration of the mechanism of interaction for the addition of (**a**) (PTEA)_50_-*b*-(PAm)_50_, (PTEA)_50_-*b*-(PAm)_200_, (**b**) (PTEA)_50_-*b*-(PAm)_1000_, and (**c**) (PTEA)_96_-*b*-(PEO)_100_ into DNA solution. (**d**) Schematic representation of the macromolecules studied: (**e**) DLS results as a function of charge ratio, at Z_+/−_ = 0.6 and 2.0, for the polyplexes studied.

The DLS data show that the size of the aggregates is around 200 nm and that after charge neutralization, precipitation occurs for the polymers containing the shorter blocks, PAm_50_ and PAm_200_. Interestingly, the same was not observed for the copolymers (PTEA)_50_-*b*-(PAm)_1000_ and (PTEA)_96_-*b*-(PEO)_100_/DNA. In fact, as illustrated in Figure 3e, the diameter of (PTEA)_96_-*b*-(PEO)_100_/DNA and (PTEA)_50_-*b*-(PAm)_1000_/DNA polyplexes is around 200 nm at Z_+/−_ = 0.6, and even at Z_+/−_ = 2.0, after charge neutralization, (PTEA)_50_-*b*-(PAm)_1000_/DNA polyplexes remained with finite size, only slightly larger, possibly due to the larger size of their neutral blocks that prevented their growth and precipitation. Similarly, for (PTEA)_96_-*b*-(PEO)_100_/DNA, the complexes also presented finite sizes although their size distribution was broader, indicating that possibly some polyplexes coacervated; but still, the aggregates remained with size in the order of 10^2^ nm, shedding light on the effect of a larger neutral block on the aggregate’s size. For (PTEA)_50_-*b*-(PAm)_50_/DNA and (PTEA)_50_-*b*-(PAm)_200_/DNA polyplexes, at Z_+/−_ = 0.6, their diameter was also around 200 nm, but at Z_+/−_ = 2.0, precipitation was observed. These results show that the size of the neutral block plays an essential role in determining the size of the polyplexes and the extent of coacervation/precipitation, as, for instance, the longer polyacrylamide block of (PTEA)_50_-*b*-(PAm)_1000_ was able to prevent its polyplex from precipitating. Figure 3 schematically illustrates the mechanism envisaged for the formation of polyplexes depending on the size and nature of their neutral blocks: the short polyacrylamide blocks eventually precipitate, whereas increasing the polyacrylamide block, the coacervates maintain almost the same size even after charge neutralization. When PEO is the neutral block, even with only 100 PEO units, finite sized coacervates are observed as remaining after charge neutralization, showing that this neutral block is more efficient in preventing the formation of precipitate.

Zeta potential data (Figure 2-IV) for titration of copolymers in DNA show the formation of negatively charged aggregates with zeta potential values around –45 mV for charge ratios up to Z_+/−_ ~ 0.8 for systems containing PAm_50_ (Figure 2a) and PAm_200_ (Figure 2b). Above that, charge inversion occurs in the range of Z_+/−_ 0.9 and 1, respectively. Differently, for the polymer PAm_1000_ (Figure 2c), these aggregates remain with small negative zeta potentials at low Z values, and at intermediate values of Z_+/−_, the aggregates reach neutrality that remains throughout the titration. This difference may be ascribed to the different PAm block sizes. For (PTEA)_96_-*b*-(PEO)_100_, the behavior is similar to the copolymers with smaller PAm blocks, where a more drastic charge inversion is observed. 

Previous studies evaluated the effect of the blocks with respect to copolymers binding to DNA, focusing on keeping the neutral block length almost constant and varying the length of the cationic block. For example, the copolymer PMAG_52_-*b*-PAEMA_63_, where PAEMA is the cationic block, binds more strongly with DNA than PMAG_56_-*b*-PAEMA_30_, which was associated with a stronger interaction due to longer cationic block [25]. Those authors also suggest that even longer neutral blocks can limit the binding with DNA, impeding the interaction of the cationic moiety with the secondary structure of DNA assuming that hydrogen bonding does not effectively participate on the formation of the complexes [25]. In the present study, where the focus is on changing the neutral block length, clearly, this was not observed, and the initial aggregate size and the energetics of binding of different (PTEA)-*b*-(PAm) with DNA are similar. Changing the neutral block to PEO leads to a small increase in the binding affinity, as revealed by the thermodynamic functions reported in Table 2.

**Figure 4:**
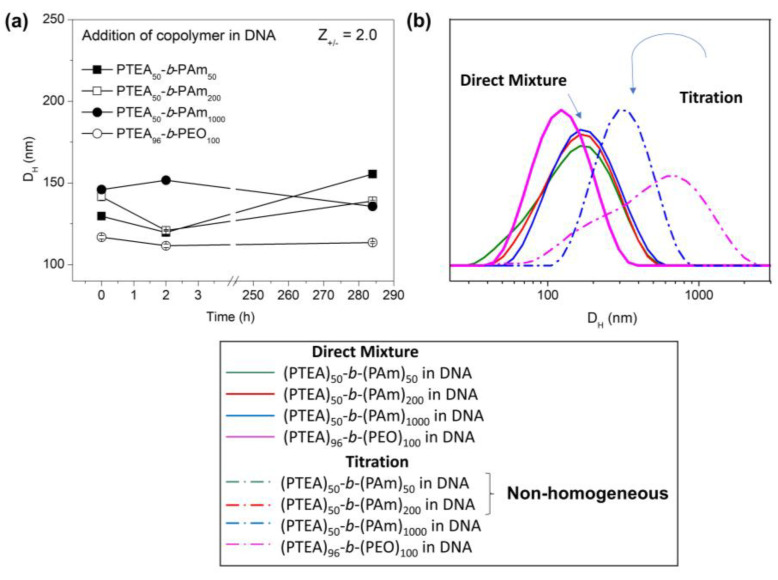
(**a**) Size of polyplexes prepared by direct mixture as function of time, with PDI values indicated as black bars and (**b**) size distribution of polyplexes obtained by direct mixture and titration, just after preparation.

To evaluate the kinetic stability of the complexes, their sizes were monitored as function of time when formed upon direct mixture of the copolymers with DNA at Z_+/−_ = 2.0. As shown in Figure 4, the complex formed between (PTEA)_96_-*b*-(PEO)_100_ and DNA displays an initial size around 117 nm, and after 2 h, it slightly decreases to 112 nm, remaining constant up to 12 days. The size for the (PTEA)_50_-*b*-(PAm)_50_ and DNA complexes was initially of 130 nm and also presented a slight decrease to 120 nm and increased slightly to 155 nm after 12 days. Both complexes of DNA and (PTEA)_50_-*b*-(PAm)_200_ or (PTEA)_50_-*b*-(PAm)_1000_ initially presented similar sizes, 141 and 146 nm, respectively. After 2 h, the (PTEA)_50_-*b*-(PAm)_200_ complex display a decrease on its size (121 nm), while (PTEA)_50_-*b*-(PAm)_1000_ and DNA complexes had a minor increase (152 nm). Interestingly, after 12 days, (PTEA)_50_-*b*-(PAm)_1000_/DNA polyplexes decrease to 136 nm, almost the same size as (PTEA)_50_-*b*-(PAm)_200_/DNA polyplexes (140 nm). Overall, the size of aggregates at Z_+/−_ = 2.0 does not vary significantly within the investigation period.

However, when we assess the effect of their preparation protocol, either by continuous titration or via direct mixing, the difference in the polyplexes size is illustrated in Figure 4b. Using the direct mixture results in polyplexes significantly smaller compared to the titration protocol. Indeed, a previous study showed the direct mixture of DTAB with diblock copolymers (PAA)_5000, g mol_^−1^-*b*-(PAm)_30,000, g mol_^−1^ generated aggregates smaller than those produced by the complex salt procedure [44]. 

**Figure 5:**
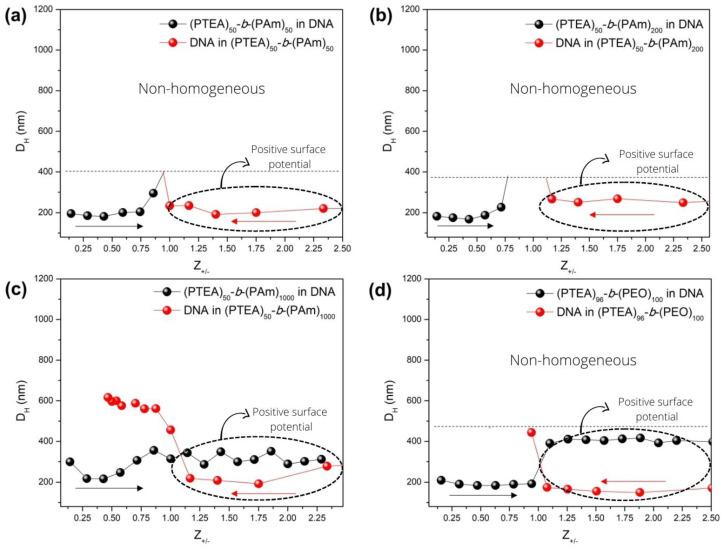
Size of polyplexes as function of charge ratio (Z_+/−_) for the addition of (PTEA)_50_-*b*-(PAm)_50_ (**a**), (PTEA)_50_-*b*-(PAm)_200_ (**b**), (PTEA)_50_-*b*-(PAm)_1000_ (**c**), and (PTEA)_96_-*b*-(PEO)_100_ (**d**) into DNA by direct addition (black) and addition of DNA into cationic block copolymer by reverse addition (red).

Polyplexes were also prepared by titrating DNA into the copolymer solution in a reverse direction as previously used, and their sizes were compared to those prepared by titrating copolymer to DNA in Figure 5. As discussed before, the direct titration, that is, the addition of copolymers (PTEA)_50_-*b*-(PAm)_50_ and (PTEA)_50_-*b*-(PAm)_200_ into DNA (curves in black), led to precipitation at Z_+/−_ approximately ≥ 1.0, while (PTEA)_50_-*b*-(PAm)_1000_ and (PTEA)_96_-*b*-(PEO)_100_ generated finite-sized structures. For the addition of DNA in the copolymers (red curves, reverse direction), at Z_+/−_ ≤ 1.0 (excess of DNA), precipitation was observed except for (PTEA)_50_-*b*-(PAm)_1000_. The delivery efficiency of a polyplex is improved whenever it possesses positive charge because it can interact with negatively charged cell membranes, and they are internalized to the cell via endocytosis [45]. After that, DNA must dissociate from the complexes to be released into the cytoplasm and conducted into the nucleus [45]. Polyplexes with approximately 200 nm displaying positive surface potential were obtained by adding DNA to all cationic copolymers. Upon the addition of (PTEA)_50_-*b*-(PAm)_1000_ to DNA, it was possible to obtain polyplexes with positive surface potential; however, the size of the formed polyplexes was in the order of 400 nm, whereas polyplexes of around 200 nm were obtained when DNA was added to (PTEA)_50_-*b*-(PAm)_1000_. This observation stresses the importance of selecting the preparation protocol for producing DNA polyplexes.

**Figure 6:**
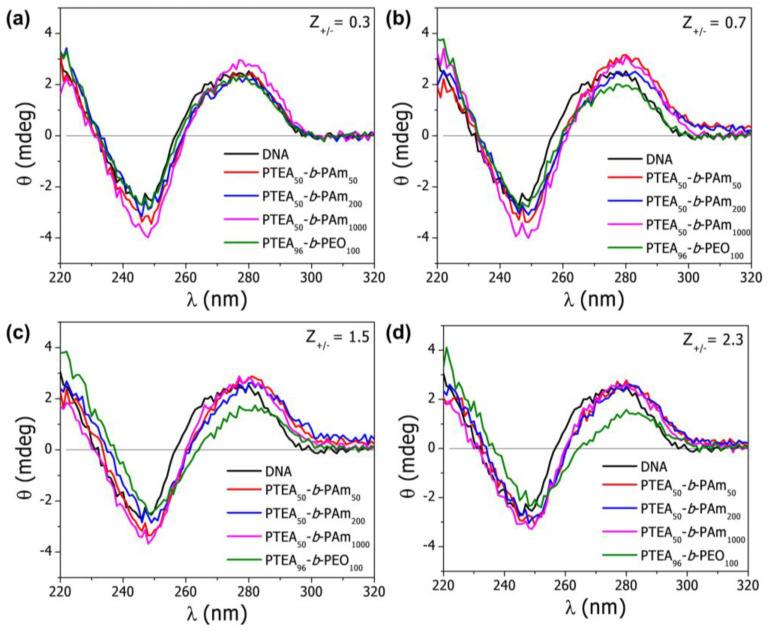
CD spectra of 0.4 mM DNA titrated with (PTEA)_50_-*b*-(PAm)_50_, (PTEA)_50_-*b*-(PAm)_200_, (PTEA)_50_-*b*-(PAm)_1000_, or (PTEA)_96_-*b*-(PEO)_100_ with DNA at (**a**) *Z*_+/-_ = 0.3, (**b**) *Z*_+/-_ = 0.7, (**c**) *Z*_+/-_ = 1.5, and (**d**) *Z*_+/-_ = 2.3.

Circular dichroism (CD) was used to evaluate whether there were changes of DNA conformation induced by the addition of the cationic copolymers. The CD spectrum of DNA solution shows a positive band near 275 nm and a negative band close to 245 nm related to the stacking of DNA bases and to the helical structure of DNA, respectively (Figure 6). Upon addition of (PTEA)-*b*-(PAm), at *Z*_+/-_ = 0.3, the DNA secondary structure is not significantly affected independently of the copolymer although a red shift of the peak position is observed at *Z*_+/-_ = 0.7 and 1.5, which is similar to that observed in previous studies [25,46]. At *Z*_+/-_ = 2.3, there is no observable change on the DNA structure, which may indicate that the interaction is mainly electrostatic considering that hydrogen-boding interaction is expected to modify the CD profile [20]. On the other hand, (PTEA)-*b*-(PEO)_100_ induces changes in the DNA secondary structure in accordance with its strongest interactions with DNA, corroborating the previously discussed ITC results.

The CD results indicate that (PTEA)_96_-*b*-(PEO)_100_ displays the strongest binding to DNA, which can be ascribed to its longer cationic block. Other than that, the neutral block seems not to be interacting with DNA through hydrogen bonding between the acrylamide groups in the PAm block and the bases in the DNA. Indeed, Prevette and co-workers studied the condensation of DNA using a series of poly(glycoamidoamine)s and concluded that the binding mechanism is not only electrostatic but also through hydrogen bonding between the groups in the carbohydrate co-monomer and the DNA base pair [14]. Nevertheless, it has been suggested that this type of interaction occurs when the DNA chain is close to the groups that are capable of interacting through hydrogen bonding. When the blocks are separated, as in diblock copolymers, and the neutral block is not close to the cationic–anionic interaction sites [25], this type of interaction might not be so effective, as in the present study. 

## 3. Materials and Methods

### 3.1. Materials

Salmon sperm DNA (2000 bp) was obtained from Sigma-Aldrich and used as received. Potassium ethyl xantogenate (purity 96%), methyl 2-bromopropionate (purity 98%), [2-(methacryloyoxy)ethyl] trimethyl ammonium chloride (TEA 80%), and 4,4′-Azobis(4-cyanovaleric acid) from Sigma-Aldrich were used without prior purification. Organic solvents methanol, hexane, ethyl acetate, isopropyl alcohol, acetone, ethyl ether, and 1,4-dioxane were purchased from Synth. Acrylamide (Am, Fluka, purity ≥ 98%) was recrystallized from methanol. The block copolymer (PTEA)-*b*-(PEO) was prepared as described below from poly(ethylene oxide)-*b*-poly(n,n-dimethylaminoethyl methacrylate), namely PEO-*b*-PDMAEMA, which was obtained from Polymer Source Inc., Dorval, QC, Canada, with M_n_ of 5000 g mol^−1^-*b*-15000 g mol^−1^. All other reagents were of the highest purity available and were used as received. Deionized water (Milli-Q, Millipore-18.2 MΩ cm) was used for all experiments.

### 3.2. Block Copolymer Synthesis

#### 3.2.1. Preparation of RAFT Agent (CTA)

The copolymers were prepared under argon atmosphere in a sealed flask by adding the RAFT agent (CTA), TEA, and initiator. The synthesis of S-(2-methylpropionate) O-ethyl xanthate (RAFT agent) was performed according to the methodology described in the literature [47], only using methanol as solvent instead of ethanol. The product was characterized by H^1^ Nuclear Magnetic Resonance (^1^H-NMR (250 MHz, CDCL_3_): d[ppm] = 4.63, 2H, q (C(S)OCH_2_), 4.38, 1H, q (CH), 3.76, 3H, s (OCH_3_), 1.58, 3H, d (CH_3_) and 1.42, 3H, t (CH_3_)). 

#### 3.2.2. Preparation of Homopolymer Poly(trimethyl-ammonium Chloride Metacriloiloxietil) and Block Copolymers PTEA-b-PAm

**Scheme 2:**
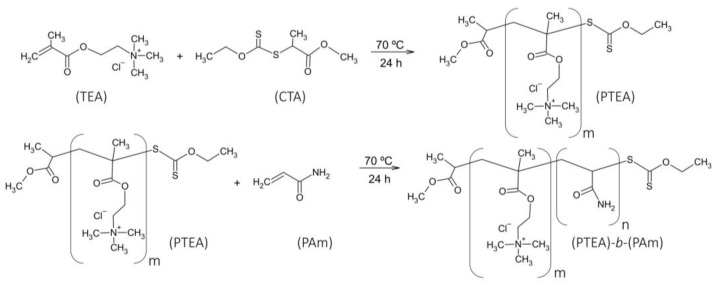
Schematic overview of the block copolymers preparation.

The block copolymers, poly(trimethyl-ammonium chloride metacriloiloxietil)-*b*-poly(acrylamide) and (PTEA)_m_-*b*-(PAm)_n_ (where m and n represent the degrees of polymerization of TEA and Am, respectively), were synthesized by Reversible Addition-Fragmentation chain Transfer (RAFT) using a xanthate as RAFT agent, following a methodology described in the literature [44,48] with some adaptations. The reactions were performed under argon atmosphere in a sealed flask by adding the transfer agent O-ethyl xanthate (CTA) and [2-(Methacryloyoxy)ethyl] trimethyl ammonium chloride (TEA) in a mixture of deionized water and isopropyl alcohol (4:1 *v*/*v*). Then, the initiator (4,4′-Azobis(4-cyanovaleric acid)) were added slowly dropwise, and the system reacted for 24 h at 70 °C (Scheme 2) [48]. For block copolymerization, acrylamide and initiator were added to the reaction and the solution left to react at 70 °C for 24 h. The copolymer was isolated from the solution by precipitation with an excess of 1,4-dioxane. Then, the copolymers were filtered and washed with 1,4-dioxane and dried at the room temperature using a vacuum [48]. The expected size of each block was defined in terms of the quantity of reactants used in the synthesis of (PTEA)-*b*-(PAm) [49], according to Table S1 (Supplementary Material).

#### 3.2.3. Modification of (PTEA)-*b*-(PEO)

**Scheme 3:**
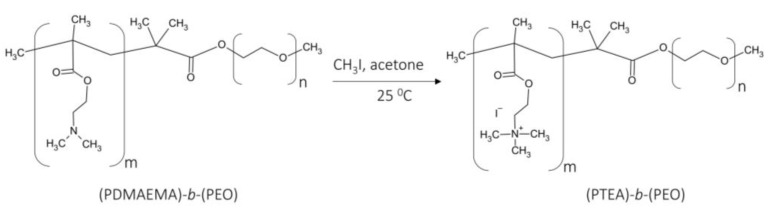
Schematic overview of (PTEA)-*b*-(PEO) synthesis.

To prepare (PTEA)-*b*-(PEO), the block copolymer (DMAEMA) was modified aiming at the quarternization of the tertiary amine groups according to the method described by Ranger and co-workers [50]. Briefly, the polymer (0.266 g) was added to a round-bottom flask with 10 mL of acetone. Then, 250 μL of methyl iodide 99% was added. The solution was stirred during 16 h at 25 °C (Scheme 3). The resulting product was dialyzed against water and freeze-dried.

### 3.3. Polyplexes Preparation

Polyplexes were prepared by titrating the 4.0 mM block copolymer solution into a 0.4 mM DNA solution until the desired mole ratio (Z_+/−_) was reached. Both solutions were prepared with 5.0 mM of NaCl. The pH of the solutions was not corrected since the charges of the polyelectrolytes are expected to be independent of the pH medium. The polyplexes were also prepared by titrating a 4.0 mM DNA solution into a 0.4 mM block copolymer solution. Further experiments were performed by adding block copolymer into DNA solution in one step to achieve the charge ratio (Z_+/−_) of 2.0, and the complexes’ sizes were monitored as a function of time. During this period of time, the complexes formed were stored at 25 °C.

### 3.4. Isothermal Titration Calorimetry

Titrations of (PTEA)-*b*-(PEO) or (PTEA)-*b*-(PAm) with DNA solutions were performed to investigate the energetics of binding. ITC experiments were performed in a VP-ITC (MicroCal, Amherst, MA, USA) isothermal titration calorimeter with a sample cell of 1.43 mL. A more concentrated solution (4.0 mM) of polymer was consecutively injected with a gastight syringe that also acted as a stirrer (at 329 rpm) into a solution at 0.4 mM of DNA in the cell. This concentration was calculated considering the molar composition in terms of charge equivalents (for this system, one charge equals one mer). Injection volumes varied between 10 and 15 μL with an interval of 800 s between each injection. The volume of the cell was kept constant during the experiments due to an overflow of solution, and this was considered during calculations of the actual cell concentrations. Polymer and DNA dilution heat effects were determined and found to be negligible.

### 3.5. Dynamic Light-Scattering and Zeta Potential Measurements

Dynamic light-scattering and zeta potential measurements were performed with a Malvern Instruments Autosizer model 4700, UK. The hydrodynamic diameter (D_H_) of the polyplexes was expressed by means of their Z-average. These polyplexes were obtained by titrating copolymer into DNA solution or vice versa and by direct mixture at a specific charge ratio. The electrophoretic mobility of the samples was determined from the average of 15 cycles of an applied electric field. Their zeta potential was determined from their electrophoretic mobility using the Smoluchowski approximation.

### 3.6. Circular Dichroism (CD)

CD spectra of the polyplexes were obtained using a Jasco 715, Japan, spectropolarimeter to monitor the conformation of DNA in the polyplexes. The spectral range varied from 220–320 nm with a scan rate of 20 nm·min^−1^, and the reported spectra are an average of three accumulations. The samples were maintained at 25 °C in a 1 mm pathlength quartz cuvette. To prepare the polyplexes, a DNA solution of 0.4 mM was used and titrated with a 4.0 mM polymer solution until the desired charge ratio. 

## 4. Conclusions

Four different block copolymers were used with the same cationic block in order to compare the influence of the nature and length of the neutral block on their interaction with salmon sperm DNA. Calorimetric measurements showed that the interaction is endothermic, around 4.0 kJ mol^−1^ of charges for the copolymers (PTEA)-*b*-(PAm) and around 5.5 kJ mol^−1^ of charges for (PTEA)_96_-*b*-(PEO)_100_, indicating that the formation of the complex is entropically driven, probably due to the release of counterions and water molecules. For all copolymers, the enthalpy change is constant up to a critical charge ratio (Z_+/−_) of about 0.8 for the addition of polymer to DNA. These calorimetric measurements also confirm that the polyplex formation is a two-step process including an ion-pairing and a coacervation stages.

The complexes hydrodynamic diameter was determined by DLS as around 200 nm before Z_+/−_ approximately 1.0. After charge neutrality, precipitation occurred for the complexes of (PTEA)_50_-*b*-(PAm)_50_ and (PTEA)_50_-*b*-(PAm)_200_ and DNA, whereas the size of polyplexes of DNA and (PTEA)_50_-*b*-(PAm)_1000_ remained almost constant even at high Z_+/−_. The polyplexes of (PTEA)_96_-*b*-(PEO)_100_ and DNA display diameters around 200 nm at Z_+/−_ = 0.6, and after charge neutrality was reached, a broad size distribution was observed. Interestingly, for all the copolymers studied, the complexes prepared by direct mixture at Z_+/−_ = 2.0 were smaller than those prepared by titration, indicating that the protocol of preparation affects the polyplexes formation. Varying the order of addition, that is, performing the titration of copolymer into DNA or vice versa (direct or reverse titration, respectively), was found to affect both the size and charge of the polyplexes, and this can be used to modulate the characteristics of polyplexes for varied applications. 

## Figures and Tables

**Table 1:** Composition of the copolymers studied.

	Number of Monomers of Cationic Block	M_w_, Cationic Block (g mol^−1^)	Number of Monomers of Neutral block	Mw, Neutral Block (g mol^−1^)	Dispersity (M_w_/M_n_)
(PTEA)_50_-*b*-(PAm)_50_	50	10,581	50	3550	1.5 *
(PTEA)_50_-*b*-(PAm)_200_	50	10,203	200	14,200	1.9 *
(PTEA)_50_-*b*-(PAm)_1000_	50	10,526	1000	71,000	2.3 *
(PDMAEMA)_96_-*b*-(PEO)_100_	96	15,000	113	5000	1.3 **
(PTEA)_96_-*b*-(PEO)_100_	96	28,695	113	5000	1.3 **

* Experimental values obtained as described in the supplementary material. ** According to the copolymer provider.

**Table 2:** Thermodynamic parameters obtained by fitting of the ITC curves for processes A and C.

Process A	$\Delta H_{b}^{A}$ **(kJ mol^−1^)**	$K_{b}$	$n_{A}$	ΔG **(kJ mol^−1^)**	ΔS **(J mol^−1^ K^−1^)**
PTEA_50_-*b*-PAm_50_ in DNA	4.2	3.1 × 10^6^	0.96	−37.0	138
PTEA_50_-*b*-PAm_200_ in DNA	4.1	2.5 × 10^7^	0.85	−42.2	155
PTEA_50_-*b*-PAm_1000_ in DNA	4.4	6.3 × 10^6^	0.83	−38.8	145
PTEA_96_-*b*-PEO_100_ in DNA	5.5	5.0 × 10^6^	0.83	−38.2	147
**Process C**	$\Delta H_{b}^{C}$ **(kJ mol^−1^)**	$K_{b}$	$n_{C}$	ΔG **(kJ mol^−1^)**	ΔS **(J mol^−1^ K^−1^)**
PTEA_50_-*b*-PAm_50_ in DNA	−1.4	6.6 × 10^5^	1.04	−33.2	107
PTEA_50_-*b*-PAm_200_ in DNA	−1.1	3.1 × 10^6^	0.96	−37.1	121
PTEA_50_-*b*-PAm_1000_ in DNA	−2.0	1.4 × 10^6^	0.92	−35.1	111
PTEA_96_-*b*-PEO_100_ in DNA	−4.3	1.4 × 10^6^	0.89	−35.0	103

* The parameters were obtained considering the molar composition in terms of charge equivalents (for this system, one charge equals one mer).

## Data Availability

The data presented in this study are available on request from the corresponding author.

## Supplementary Materials

The following supporting information can be downloaded at: https://www.mdpi.com/article/10.3390/molecules28010398/s1, Figure S1: Gel permeation chromatograms obtained for the copolymers (PTEA)_50_-*b*-(PAm)_50_, (PTEA)_50_-*b*-(PAm)_200_, and (PTEA)_50_-*b*-(PAm)_1000_; Table S1: Mass of reactants used in the synthesis of copolymers (PTEA)_50_-*b*-(PAm)_50_, (PTEA)_50_-*b*-(PAm)_200_, and (PTEA)_50_-*b*-(PAm)_1000_ [49].

## Sample Availability

Samples of the compounds are available from the authors.
