# Supplementary material for: Assessing the Contribution of the Neutral Blocks in DNA/Block-Copolymer Polyplexes: Poly(acrylamide) vs. Poly(ethylene Oxide)
Source: Molecules. 2023 Jan 3;28(1):398. doi: 10.3390/molecules28010398 (PMC9824764; doi:10.3390/molecules28010398)
Supplement: Supplementary file 1 [file molecules-28-00398-s001.zip › molecules-2118800-supplementary.pdf]

Supplementary Material

Assessing the contribution of the neutral blocks in DNA/block-copolymer polyplexes: poly(acrylamide) vs poly(ethylene oxide)

Renata Mello Giona, Letícia Vitorazi and Watson Loh*

*wloh@unicamp.br

SI.1 - Polymer characterization - dispersity

For the characterization of the copolymers, the dispersity (DI) was determined by gel permeation chromatography (GPC). The equipment used was a Viscotek GPC max VE 2001 equipped with 3 SB-806M HQ columns. The calibration curve was performed with low-dispersion poly (ethylene oxide), PEO, standards in the molar mass range of 630 to 584.000 g mol^{-1}. The measurements were made in an aqueous $NaSO_4$ solution at 0.05 mol L^{-1}, with a flow rate of 1 mL min^{-1}, using the refractive index detector. The sample concentration was approximately 8 $mgmL^{-1}$, with an injection volume of 100 μL.

The gel permeation chromatograms obtained for the copolymers are shown in Figure S1. The main signal obtained in each chromatogram presented retention volumes of 28.55; 26.90 and 25.24, respectively for the copolymers $PTEA_{50}$-*b*-PAm_{50}, $PTEA_{50}$-*b*-PAm_{200} and $PTEA_{50}$-*b*-PAm_{1000}. These values are related to molar masses of M_n = 3,811 and M_w = 5,560 g mol^{-1} (DI: 1.5); M_n = 14,868 and M_w = 27,897 g mol^{-1} (DI: 1.9) and M_n = 78,549 and M_w = 184,199 g mol^{-1} (DI: 2.3), respectively.

In this study, only DI was considered since the polymer injected in the calibration curve was PEO. The molar mass considered is based on the amount used in the synthesis described in the main text of the article.

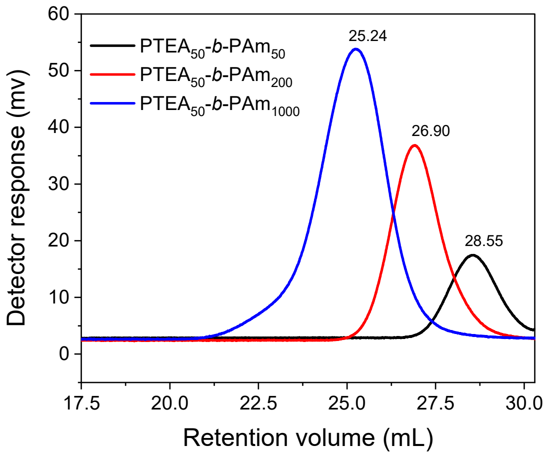

Figure S1. Gel permeation chromatograms obtained for the copolymers $(PTEA)_{50}$-*b*-$(PAm)_{50}$, $(PTEA)_{50}$-*b*-$(PAm)_{200}$, and $(PTEA)_{50}$-*b*-$(PAm)_{1000}$.

SI.2 – Block copolymer synthesis

The block copolymers, poly(trimethyl-ammonium chloride metacriloilox-ietil)-b-poly(acrylamide), $(PTEA)_m$-*b*-$(PAm)_n$ (where m and n represent the degrees of polymerization of TEA and Am, respectively) were synthesized by Reversible Addition-Fragmentation chain Transfer (RAFT) using xanthate as RAFT agent. The expected size of each block ($\bar{M}_{n_{\text{Theory}}}$) was defined in terms of the mass of reactants (Table S1) used in the synthesis of (PTEA)-*b*-(PAm), according to Eq. 1 [1].

$$\bar{M}_{n_{\text{Theory}}} = \frac{[\text{monomer}]_0 \cdot M_{w\text{monomer}} \cdot \rho}{[CTA]_0} + M_{wCTA} \quad (1)$$

where $[\text{monomer}]_0$ is the initial monomer concentration, ρ is the fractional conversion, $M_{wmonomer}$ is the molecular weight of the monomer, M_{wCTA} is the molecular weight of the RAFT agent, and $[CTA]_0$ is the initial concentration of the RAFT agent.

Table S1. Mass of reactants used in the synthesis of copolymers $(PTEA)_{50}$-*b*-$(PAm)_{50}$, $(PTEA)_{50}$-*b*-$(PAm)_{200}$, and $(PTEA)_{50}$-*b*-$(PAm)_{1000}$.

	Mass (g)		
	$(PTEA)_{50}$-*b*-$(PAm)_{50}$	$(PTEA)_{50}$-*b*-$(PAm)_{200}$	$(PTEA)_{50}$-*b*-$(PAm)_{1000}$
TEA, 80%	12.009	11.996	11.980
CTA	0.1926	0.1988	0.1935
Am	3.2810	13.1363	65.600

SI.3 – References

49. Lowe, A.B.; McCormick, C.L. Reversible Addition-Fragmentation Chain Transfer (RAFT) Radical Polymerization and the Synthesis of Water-Soluble (Co)Polymers under Homogeneous Conditions in Organic and Aqueous Media. *Progress in Polymer Science (Oxford)* 2007, *32*, 283–351.
